# Relationship between mobility, violence and HIV/STI among female sex workers in Andhra Pradesh, India

**DOI:** 10.1186/1471-2458-12-764

**Published:** 2012-09-11

**Authors:** Sowmya Ramesh, Deepika Ganju, Bidhubhusan Mahapatra, Ram Manohar Mishra, Niranjan Saggurti

**Affiliations:** 1 Population Council, 1st Floor, 142 Golf Links, New Delhi, 110003, India

**Keywords:** HIV, Mobility, Violence, Sex work, Risky behaviour

## Abstract

**Background:**

Violence and mobility have been identified as critical factors contributing to the spread of HIV worldwide. This study aimed to assess the independent and combined associations of mobility and violence with sexual risk behaviors and HIV, STI prevalence among female sex workers (FSWs) in India.

**Methods:**

Data were drawn from a cross-sectional, bio-behavioral survey conducted among 2042 FSWs across five districts of southern India in 2005–06. Regression models were used to estimate odds ratios and 95% confidence intervals (CIs) for sexual risk behaviors and HIV infection based on experience of violence and mobility after adjusting for socio-demographic and sex work related characteristics.

**Results:**

One-fifth of FSWs (19%) reported experiencing violence; 68% reported travelling outside their current place of residence at least once in the past year and practicing sex work during their visit. Mobile FSWs were more likely to report violence compared to their counterparts (23% vs. 10%, p < 0.001). Approximately 1 in 5 tested positive for HIV. In adjusted models, FSWs reporting both mobility and violence as compared to their counterparts were more likely to be infected with HIV (Adjusted odds ratio (adjusted OR): 2.07, 95% CI: 1.42–3.03) and to report unprotected sex with occasional (adjusted OR: 2.86, 95% CI: 1.76–4.65) and regular clients (adjusted OR: 2.07, 95% CI: 1.40–3.06).

**Conclusions:**

The findings indicate that mobility and violence were independently associated with HIV infection. Notably, the combined effect of mobility and violence posed greater HIV risk than their independent effect. These results point to the need for the provision of an enabling environment and safe spaces for FSWs who are mobile, to augment existing efforts to reduce the spread of HIV/AIDS.

## Background

Violence and mobility are increasingly being recognized as important risk factors contributing to the spread of HIV and sexually transmitted infection (STI) worldwide [1-11]. High rates of violence perpetrated against female sex workers (FSWs) have been consistently documented in developing countries [12-14]. In a study of mobile FSWs (those who travelled to two or more places for sex work over a two-year period) in India, approximately one-third reported experiencing violence [14]. Both in India and elsewhere, published literature indicates that FSWs exposed to violence were more likely to be infected with HIV and other STI than those who did not report such experiences [15,16]. Forced unprotected sexual encounters were described as the most likely cause for their heightened vulnerability to HIV [12,14,17]. Further, research indicates that forced sex was a barrier to condom negotiation [12] and increased the likelihood of condom failure [13,18].

In addition to violence, recent research has identified employment-related mobility (intra-district, inter-district, or inter-state) as another major risk factor associated with HIV [4,8-10,19-23]. In India, as is the case globally, FSWs are highly mobile for sex work [5] due to police harassment [24]; to escape stigma and discrimination [4,25]; and to attract a different or wider client base [11]. Research suggests that FSWs who were mobile for sex work had higher rates of HIV as compared to non-mobile FSWs [5,10], possible reasons being their experience of violence [11,14,17], sexual risk behaviors such as unprotected sex [4,5,25], lack of access to condoms [25], inability to negotiate safe sexual practices [26], and limited access to health care services [11,27] and HIV prevention programs [11,26] in new locations.

While studies have demonstrated the independent effect of mobility and experience of violence on FSWs’ sexual risk behaviors and STI, there is a paucity of literature on the combined relationship of mobility and violence on HIV risk behaviors, STI and HIV. This paper seeks to address this gap in the literature by examining: (a) the independent association between mobility for sex work and violence; and (b) the independent as well as the combined association of mobility for sex work and violence on risky sexual behaviors and STI, including HIV.

## Methods

### Design, setting and sample

Data were drawn from the Integrated Behavioral and Biological Assessment (IBBA), a cross-sectional survey conducted in 2005–06 among FSWs in eight high HIV prevalence districts of Andhra Pradesh state, India (Chitoor, Guntur, East Godavari, Prakasam, Hyderabad, Karim Nagar, Warangal and Visakhapatnam) [28]. A probability sampling method was adopted using two different approaches: (1) conventional cluster sampling for brothel-based and home-based sex workers, and (2) conventional time-location cluster sampling for street-based FSWs. The overall survey design including district selection, sample size calculation and participant recruitment has been described in detail elsewhere [29].

Overall, 3271 FSWs completed the behavioral interview and provided biological (blood and urine) samples in Andhra Pradesh. Of the eight districts surveyed, data from three districts, namely Hyderabad, Karim Nagar and Warangal, were not included in the current analysis, as a different questionnaire was used in these districts, which did not include mobility-related questions; this resulted in an analytical sample of 2042 FSWs.

Face-to-face interviews were conducted by trained field workers in the local language, Telugu, using a structured questionnaire that included questions on socio-demographic characteristics, sexual behavior, mobility and experience of violence. Interviews were conducted in locations previously hired for data collection purposes; respondents were escorted by members of the field team from solicitation sites to the interview location. In addition, biological samples were tested for HIV and other STI, including *Chlamydia trachomatis* (chlamydia), *Neisseria gonorrhea* (gonorrhea), syphilis and Herpes Simplex Virus-type 2 (HSV–2). The testing procedures adopted in the survey have been described in detail elsewhere [29].

### Ethical considerations

Ethical clearances were obtained prior to the survey. Statutory approval for conducting the IBBA and its protocols was obtained from the Government of India’s Health Ministry Screening Committee. A comprehensive consent process was adopted: respondents were first informed in detail about all aspects of the survey, following which oral consent was separately obtained for the behavioral and biological components.

### Measures

#### Socio-demographic and sex work characteristics

The socio-demographic characteristics of FSWs considered in this paper were based on single items in the questionnaire, which included age (<30, 30+), literacy (illiterate, literate), marital status (never, currently or previously married), alcohol consumption (ever, never), primary place where clients were entertained (home including rented room; brothel including *dhaba*, bar/night club and lodge; and public place including park, street, cinema hall, bus stand, railway station and vehicle), having a regular non-paying partner and client volume per week (<10, 10+). Duration of sex work was computed by subtracting the respondent’s age at initiation of commercial sex from her age at the time of interview. Both socio-demographic and sex work related characteristics were used as covariates in the multivariate analyses.

#### Violence and mobility for sex work

Experience of violence and mobility for sex work were the two key independent measures used in this paper. Respondents were classified as having experienced violence based on responses to the following question: whether they had been beaten or physically forced by any individual to have sexual intercourse against their will in the past one year. Similarly, respondents were classified as mobile for sex work if they had travelled outside their current place of residence in the past one year and practiced sex work during their visit. These two variables were combined to create four categories in order to examine the combined associations of violence and mobility for sex work: (i) no violence and not mobile; (ii) no violence but mobile; (iii) violence but not mobile; and (iv) both violence and mobility.

#### Sexual risk behaviors, HIV and STI

The dependent measures used in this paper were: sexual risk behaviors, STI and HIV infection. Sexual risk behaviors were measured using two key variables that determined FSWs’ unsafe sex behavior: (1) no condom use at most recent sex with occasional clients; and (2) no condom use at most recent sex with regular clients.

Information on STI and HIV was based on the laboratory test results of biological samples. Participants were considered HIV-positive if their blood samples tested positive on the Microelisa test, and were confirmed by the Genedia HIV ½ ELISA 3.0 test. Participants were considered positive for chlamydia and gonorrhea if the infection was detected in their urine samples by the nucleic acid amplification test (NAAT). In this study, participants were considered to be infected with an STI if they were diagnosed with chlamydia or gonorrhea. The syphilis and HSV–2 test results were not included in the analysis as the syphilis test results may not reflect current infection and the HSV–2 test was conducted among only 10% of those who provided biological samples.

### Statistical analyses

Sample characteristics were assessed to identify the factors that differed by mobility status and experience of violence, using chi-square contingency tables. Logistic regression models were used to estimate odds ratios (OR) and corresponding 95% confidence intervals (CI) to analyze the relationship between reported violence, mobility for sex work, and sexual risk behaviors and STI including HIV. A series of multivariate logistic regression models were constructed to measure: (i) the association between mobility for sex work and violence and vice versa; (ii) the association between mobility for sex work and sexual risk behaviors, HIV and STI status; (iii) the association between violence and sexual risk behaviors, HIV and STI status; and (iv) the combined association of mobility for sex work and the experience of violence on sexual risk behaviors, HIV and STI status. Adjusted OR and CI are presented. Sampling weights were used to account for the differential recruitment of FSWs by typology within districts, differential probabilities of selection across districts and differential non-response rates. The weighting methodology has been described elsewhere [29]. Statistical analyses were performed using STATA version 11.1.

## Results

### Socio-demographic and sex work characteristics

Of the 2042 FSWs included in the analyses, one-fifth (19%) had experienced violence and two-thirds (68%) had travelled outside their current place of residence at least once in the past year and practiced sex work during their visit (Table 1). Experience of violence was high among FSWs who were previously married, had ever consumed alcohol, had entertained clients in a public place, were mobile for sex work and had been engaged in sex work for more than four years. Similarly, mobility for sex work was high among FSWs who were previously married, had ever consumed alcohol, experienced violence, practiced sex work for more than four years, had a higher client volume per week and had a regular non-paying partner.

**Table 1:** Background characteristics of female sex workers by experience of violence and mobility status in Andhra Pradesh, India

**Characteristics**	**Total sample (%)**	**Experienced violence in the past one year**^**a**^			**Mobility for sex work in the past one year**^**b**^		
		**No (%)**	**Yes (%)**	**p-value**	**No (%)**	**Yes (%)**	**p-value**
**Total sample**	**2042**	**1690**	**352**		**714**	**1328**	
**Age** (in years)							
<30	45.1	81.0	19.0	0.718	31.1	68.9	0.344
30+	54.9	81.6	19.4		33.1	66.9	
**Marital status**							
Currently married	67.0	83.2	16.8	0.006	33.2	66.8	0.019
Never married	6.7	80.7	19.3		39.0	61.0	
Previously married	26.3	76.8	23.2		28.0	72.0	
**Literacy**							
Illiterate	60.2	82.3	17.7	0.177	33.7	66.3	0.068
Literate	39.8	79.9	20.1		29.9	70.1	
**Alcohol consumption**							
Never	30.2	90.0	10.0	<0.001	53.3	46.7	<0.001
Ever	69.8	78.0	22.0		22.9	77.1	
**Experienced violence in the past one year**^a^							
No	81.3	-	-	-	35.6	64.4	<0.001
Yes	18.7				17.6	82.5	
**Mobility for sex work in the past one year**^b^							
No	32.2	89.8	10.2	<0.001	-	-	-
Yes	67.8	77.3	22.7				
**Duration of sex work** (in years)							
< 5	45.7	84.2	15.8	0.003	35.2	64.8	0.01
5+	54.3	79.0	21.0		29.7	70.3	
**Have a regular non-paying partner**							
No	24.9	80.5	19.5	0.580	41.4	58.6	<0.001
Yes	75.1	81.6	18.4		29.2	70.8	
**Primary place for entertaining clients**^c^							
Home-based	51.8	84.3	15.7	<0.001	33.3	66.7	0.182
Brothel-based	32.2	81.0	19.0		29.5	70.5	
Public place	16.0	72.6	27.4		34.2	65.8	
**Client volume per week**							
<10	48.0	82.7	17.3	0.121	40.8	59.2	<0.001
10+	52.0	80.0	20.0		23.8	76.2	

^a^ Physically beaten or forced to have sexual intercourse by any individual against their will in past one year.

^b^ Travelled outside their current place of residence and practiced sex work during their visit in past one year.

^c^ Home-based includes home and rented room; brothel-based includes brothel, *dhaba*, bar/night club and lodge; public place includes park, street, cinema hall, bus stand, railway station and vehicle.

### Association between violence, mobility for sex work and sexual risk behaviors

Sexual risk behaviors were significantly associated with experience of violence (Table 2). FSWs experiencing violence were more likely to report no condom use in their most recent sexual encounter with occasional clients (adjusted OR: 2.23, 95% CI: 1.57–3.18) and regular clients (adjusted OR: 1.64, 95% CI: 1.22–2.20) than those who did not report such experiences.

**Table 2:** Association between violence, mobility for sex work and sexual risk behaviors among female sex workers in Andhra Pradesh, India

	**No condom use at most recent sex with occasional clients**			**No condom use at most recent sex with regular clients**		
	**% (N)**	**adjusted OR**^**a**^**(95%CI)**	**adjusted OR**^**b**^**(95%CI)**	**% (N)**	**adjusted OR**^**a**^**(95%CI)**	**adjusted OR**^**b**^**(95%CI)**
**Experience of violence**^c^						
No	7.4 (1635)	Referent	-	14.8 (1643)	Referent	-
Yes	16.0 (345)	2.23 (1.57, 3.18)***	-	22.1 (345)	1.64 (1.22, 2.20)*	-
**Mobility for sex work**^d^						
No	6.8 (677)	Referent	-	13.2 (687)	Referent	-
Yes	10.0 (1303)	1.29 (0.88, 1.91)	-	17.5 (1301)	1.26 (0.94, 1.69)	-
**Experience of violence and mobility**^e^						
No violence and not mobile	6.4 (610)	-	Referent	12.8(618)	-	Referent
No violence but mobile	7.9 (1025)	-	1.21 (0.80, 1.87)	15.9 (1025)	-	1.24 (0.90, 1.71)
Violence but not mobile	11.3 (68)	-	1.73 (0.72, 4.12)	17.1 (69)	-	1.52 (0.75, 3.11)
Both violence and mobility	16.9 (277)	-	2.86 (1.76, 4.65)***	23.1(276)	-	2.07 (1.40, 3.06)***

OR: Odds ratio; CI: Confidence interval.

^a^ Model adjusted for age, marital status, literacy, alcohol consumption, duration of sex work, primary place of entertaining clients, client volume per week, having a regular male partner and experience of sexual violence in past year/mobility for sex work in past year.

^b^ Model adjusted for age, marital status, literacy, alcohol consumption, duration of sex work, primary place of entertaining clients, client volume per week and having a regular male partner.

^c^ Physically beaten or forced to have sexual intercourse by any individual against their will in past one year.

^d^ Travelled outside their current place of residence and practiced sex work during their visit in past one year.

^e^ No violence and not mobile: was not physically beaten or forced to have sexual intercourse against the will and did not travel and practice sex work during their visit outside their current place of residence in past one year; no violence but mobile: was not physically beaten or forced to have sexual intercourse against their will but travelled and practiced sex work during their visit outside their current place of residence in past one year; violence but not mobile: was physically beaten or forced to have sexual intercourse against the will but did not travel and practice sex work during their visit outside their current place of residence in past one year; both violence and mobility: was physically beaten or forced to have sexual intercourse against the will and travelled and practiced sex work during their visit outside their current place of residence in past one year.

*p < 0.05, ** p < 0.01, *** p < 0.001.

Further, the odds of no condom use in their most recent sex with occasional clients were three times higher among FSWs who reported both mobility and violence than those who were neither mobile nor reported violence (adjusted OR: 2.86, 95% CI: 1.76–4.65). Similarly, mobile FSWs who experienced violence were significantly more likely to report no condom use in their most recent sexual encounter with regular clients than non-mobile FSWs who did not experience violence (adjusted OR: 2.07, 95% CI: 1.40–3.06).

### Association between violence, mobility for sex work and HIV/STI

Results indicate that the experience of violence and mobility for sex work were independently associated with HIV infection (Table 3). The odds of being infected with HIV were higher among FSWs who reported being beaten or raped by any individual at least once in the past year than others (adjusted OR: 1.58, 95% CI: 1.20–2.09). Similarly, compared to non-mobile FSWs, those who reported mobility for sex work were 32% more likely to be infected with HIV (adjusted OR: 1.32, 95% CI: 1.01–1.74). Further, compared to FSWs who were not mobile and did not experience violence, those who were both mobile and reported violence were two times more likely to be diagnosed as HIV-positive (adjusted OR: 2.07, 95% CI: 1.42–3.03).

**Table 3:** Association between violence, mobility for sex work and HIV/STI among female sex workers in Andhra Pradesh, India

	**Currently infected with STI**^a^			**Infected with HIV**		
	**%**	**adjusted OR**^**b**^**(95%CI)**	**adjusted OR**^**c**^**(95%CI)**	**%**	**adjusted OR**^**b**^**(95%CI)**	**adjusted OR**^**c**^**(95%CI)**
**Experience of violence**^d^						
No (N = 1690)	3.9	Referent	-	16.6	Referent	-
Yes (N = 352)	4.8	1.31 (0.78,2.29)	-	25.6	1.58 (1.20, 2.09)**	-
**Mobility for sex work**^e^						
No (N = 714)	4.10	Referent	-	14.8	Referent	-
Yes (N = 1328)	4.17	1.10 (0.66, 1.83)	-	20.0	1.32 (1.01, 1.74)*	-
**Experience of violence and mobility**^f^						
No violence and not mobile (N = 643)	4.0	-	Referent	13.4	-	Referent
No violence but mobile (N = 1047)	4.3	-	1.03 (0.60, 1.77)	18.4	-	1.43 (1.06, 1.94)*
Violence but not mobile (N = 71)	4.0	-	1.00 (0.17, 3.04)	27.6	-	2.27 (1.34, 4.16)**
Both violence and mobility (N = 281)	5.1	-	1.45 (0.72, 2.92)	25.2	-	2.07 (1.42, 3.03)***

OR: Odds ratio; CI: Confidence interval.

^a^ STI includes: chlamydia and/or gonorrhea.

^b^ Model adjusted for age, marital status, literacy, alcohol consumption, duration of sex work, primary place of entertaining clients, client volume per week, having a regular male partner and experience of sexual violence in past year/mobility for sex work in past year.

^c^ Model adjusted for age, marital status, literacy, alcohol consumption, duration of sex work, primary place of entertaining clients, client volume per week and having a regular male partner.

^d^ Physically beaten or forced to have sexual intercourse by any individual against their will in past one year.

^e^ Travelled outside their current place of residence and practiced sex work during their visit in past one year.

^f^ No violence and not mobile: was not physically beaten or forced to have sexual intercourse against the will and did not travel and practice sex work during their visit outside their current place of residence in past one year; no violence but mobile: was not physically beaten or forced to have sexual intercourse against their will but travelled and practiced sex work during their visit outside their current place of residence in past one year; violence but not mobile: was physically beaten or forced to have sexual intercourse against the will but did not travel and practice sex work during their visit outside their current place of residence in past one year; both violence and mobility: was physically beaten or forced to have sexual intercourse against the will and travelled and practiced sex work during their visit outside their current place of residence in past one year.

*p < 0.05, ** p < 0.01, *** p < 0.001.

## Discussion

Our findings indicate that a large percentage of FSWs travelled outside their current place of residence and practiced sex work during these visits, and nearly one-fifth experienced violence in this high HIV prevalence state of southern India; these results are similar to prior findings in India [5,14,17]. Additionally, the present study documents that mobile FSWs who experienced violence were two times more likely to have been diagnosed with HIV compared to those who reported neither mobility nor violence. A possible reason for the observed high prevalence of HIV among this subgroup of FSWs could be their risky sexual behaviors, as evidenced in the current study.

Consistent with previous research, our study shows that mobile FSWs were more likely to be infected with HIV than those who were not mobile [5,10,30]. While mobility for sex work per se may not be directly associated with HIV, as seen in prior research, mobility may increase FSWs’ vulnerability to exploitation and abuse as a result of operating in new environments with unknown clients and the lack of community ties for social support [11]. Empirical research suggests that mobility for sex work among FSWs is common in India and around the world [4,5,10,11,23,31], and our study further suggests that some sub-groups of FSWs were more likely to be mobile than others. For example, mobility was higher among FSWs who were currently or previously married or who had a regular non-paying partner than others; reasons for higher mobility among this sub-group could be to work in an environment of anonymity and to keep their sex worker identity separate from their private life [25]. Following their relocation to new areas, FSWs face several kinds of vulnerabilities including physical and sexual violence [11,14], a finding also observed in our study, which indicates that a greater proportion of mobile FSWs were abused as compared to those who were not mobile.

The current research also indicates that one-fifth of FSWs had experienced violence in the past year, and the experience of violence was higher in selected groups; for example, those who were previously married. Further, a higher proportion of FSWs who had experienced violence were infected with HIV than their counterparts; a finding that is consistent with previous research [15,16]. As described earlier, the underlying reasons for these FSWs’ heightened vulnerability to HIV could be multiple, such as the experience of forced sex, which may pose barriers to adopting safe sex behaviours [32]. Indeed, consistent with findings from prior research [13,14,17], we found that FSWs who experienced violence were less likely to report condom use with clients as compared to those who did not experience violence.

While there is growing recognition of the effect of mobility and violence individually on the health of FSWs, including their vulnerability to HIV [1-4,7,33,34], this study, to our knowledge, is amongst the first to assess the combined effect of mobility and violence on sexual risk behaviors and STI, including HIV, among FSWs. The prevalence of HIV increased twofold among respondents who reported both mobility for sex work and violence, compared to those who reported neither. Although not statistically significant, a higher proportion of mobile FSWs who experienced violence were infected with STI as compared to those who were neither mobile nor reported violence. This lack of significant association could be because of the low prevalence of STI diagnosed among this group of FSWs. Additionally, infections that occurred as a result of violence may have been treated as there is indiscriminate use of antimicrobials in India due to the easy availability of drugs over-the-counter without a medical prescription [35].

While this study underlines the strong association between violence, mobility and the prevalence of HIV among FSWs, the results should be interpreted with caution in light of certain limitations. First, the key independent variables considered in this study were based on self-reported responses, and the limitations of self-reported data are widely recognized [36]. Moreover, violence may have been underreported perhaps due to the stigma attached with reporting of violence or the sex workers’ perception of reporting based on only severity of violence [36]. However, the use of trained and experienced research staff while conducting the IBBA may have increased respondents’ comfort level at the time of interview and reduced underreporting. Second, in the multivariate analyses we have only accounted for factors that were measured in the survey; therefore, the associations of key independent and dependent measures could have been affected by omission variable bias. Third, while our study analyzes recent mobility status and experience of violence, the HIV seropositivity data reflect only prevalence. Therefore we cannot determine whether there is any temporal relationship between violence, mobility and HIV infection. However, this study is based on the assumption that FSWs who reported experience of violence and mobility in the recent past may have also experienced similar vulnerabilities since their entry into the sex work. Finally, the findings of this study cannot be generalized to all FSWs across India as sex work in India is complex in nature and characterized by inter- and intra-regional differences. For example, in the north Indian states, the sex work industry is relatively visible, and is largely brothel-based, whereas in the southern states a significant proportion of sex work is home-based or street-based [37-39]. However, the study results can be generalized to other geographical areas with similar sex work settings, volume of mobility and HIV prevalence. Nonetheless, these limitations do not compromise the internal validity of the data: our findings are consistent with the results of previous studies that have assessed the association between violence, mobility and sexual risk behaviors/HIV and advance the knowledge on the inter-linkages between these risk factors and sexual risk behaviors/HIV. However, future research could provide critical information on several key issues that would have implications for HIV programming. For example, studies that include temporal data could provide insights on the causal relation between mobility and violence; that is, whether mobility among FSWs leads to the experience of violence or vice versa, so that programmatically FSWs most vulnerable could be addressed through appropriate structural interventions. Additionally, studies could explore the extent to which FSWs’ degree of mobility (less mobile versus more mobile) and exposure to violence are associated with sexual risk taking behaviour and HIV.

Our finding that mobile FSWs who have experienced violence are particularly vulnerable to HIV has significant implications for the design of HIV prevention programs. To reach FSWs with different vulnerabilities, interventions would need to implement strategies that recognize and address both issues of violence and mobility among FSWs. Moreover, intervention programs need to recognize that as mobile FSWs have recently moved to new places for sex work, they may be poorly informed about HIV prevention support and service programs in the new area, and additional efforts would be required to connect them to suitable local services, such as the availability of crisis response systems that provide appropriate information and timely services to address violence.

## Conclusions

In the context of the study finding that mobile FSWs who also experience violence are at greater risk of acquiring HIV than others, special efforts are needed to address the vulnerabilities of this subgroup of sex workers. Ongoing and future programs need to explore the ways in which they can improve accessibility to support structures and services for sex workers on the move. Additionally, the ongoing efforts of community mobilization [40,41] need to be expanded to create an enabling environment and safe spaces for FSWs from perpetrators of violence. Further, it would be important to identify all FSWs who move to different places for sex work and orient them to prevention and crisis response services in the new location, which would help augment existing efforts to reduce the spread of HIV in India and elsewhere.

## Competing interests

The author(s) declare that they have no competing interests.

## Authors’ contributions

SR led conceptualization, conducted all analyses, and wrote the manuscript. DG assisted with writing and editing of the paper. BM and RMM assisted with the analyses. NS assisted with conceptualization of analytic approach and interpretation of study findings. All authors have read and approved this final submitted manuscript.

## Pre-publication history

The pre-publication history for this paper can be accessed here:

http://www.biomedcentral.com/1471-2458/12/764/prepub
